# Tracheal replacement with aortic grafts: Bench to clinical practice

**DOI:** 10.1016/j.reth.2023.09.004

**Published:** 2023-09-14

**Authors:** Shixiong Wei, Bo Yang, Taiyu Bi, Wenyu Zhang, He Sun, Yongsheng Cui, Guanghu Li, Anling Zhang

**Affiliations:** a The Department of Thoracic Surgery, The First Hospital of Jilin University, Changchun, Jilin Province, 130021, China; b The Department of Hepatobiliary and Pancreatic Surgery, General Surgery Center, The First Hospital of Jilin University, Changchun, Jilin Province, 130021, China; c The Department of Maxillofacial Surgery, Jilin FAW General Hospital, Changchun, Jilin Province, 130000, China

**Keywords:** Airway replacement, Aortic graft, Surgical technique, Tissue engineering, Mesenchymal stem cells

## Abstract

Tracheal reconstruction following extensive resection for malignant or benign lesions remains a major challenge in thoracic surgery. Numerous studies have attempted to identify the optimal tracheal replacement with different biological or prosthetic materials, such as various homologous and autologous tissues, with no encouraging outcomes. Recently, a few clinical studies reported attaining favorable outcomes using *in vitro* or stem cell-based airway engineering and also with tracheal allograft implantation following heterotopic revascularization. However, none of the relevant studies offered a standardized technology for airway replacement. In 1997, a novel approach to airway reconstruction was proposed, which involved using aortic grafts as the biological matrix. Studies on animal models reported achieving *in-vivo* cartilage and epithelial regeneration using this approach. These encouraging results inspired the subsequent application of cryopreserved aortic allografts in humans for the first time. Cryopreserved aortic allografts offered further advantages, such as easy availability in tissue banks and no requirement for immunosuppressive treatments. Currently, stented aortic matrix-based airway replacement has emerged as a standard approach, and its effectiveness was also verified in the recently reported TRITON-01 study. In this context, the present review aims to summarize the current status of the application of aortic grafts in tracheal replacement, including the latest advancements in experimental and clinical practice.

## 1 Introduction

Tracheal surgery was prohibited prior to 1960, considering the difficulty of perioperative ventilation and low cartilage healing ability. Eventually, the “2-cm Belsey rule” was accepted, according to which it was not possible to remove over 2 cm of the trachea using primary reconstruction [1]. The clinical and experimental results reported by Hermes C Grillo from Boston, together with the findings of other surgeons worldwide in the last five decades, provided resolutions to the above issues and challenges encountered in tracheal surgery, leading to the establishment of a standardized method for tracheal surgery [2,3]. The currently accepted safe extent of tracheal resection is 1/2 of the total tracheal length in adults and 1/3rd of the tracheal length in the pediatric population. In addition, studies have reported a few approaches for carinal and laryngotracheal reconstruction or resection [4].

While most of the above-stated challenges were resolved, one remained –in the surgery for extensive lesions, tracheal resection plus primary anastomosis is either not possible or induces high prevalence and mortality rates. Tracheal replacement is, however, required for the surgical treatment of extensive primary tracheal tumors including adenoid cystic carcinomas. While currently, such cases are managed through palliative treatment using T-tube, stents, or radiation, tracheal replacement becomes necessary for tracheal reconstruction following extensive lesion resection. Advancements in the field of medicine have enabled the successful replacement of even complicated organs. However, no success has been reported so far in the replacement of the trachea, which is just a simple duct for air passage in the human respiratory system [4,5]. Therefore, tracheal replacement remains a major challenge in both biological and surgical scenarios.

## 2 Five major techniques used for airway replacement

Great advancements in the field of medicine in the last five decades have enabled the successful transplantation of several complicated human organs. However, the replacement of the tracheobronchial tree, which has a considerably simple structure and function of the air passage in humans, remains to be achieved with success. In 2004, Grillo categorized different techniques for tracheal replacement and reconstruction into the following five types – foreign materials, non-viable tissues, autogenous tissues, tracheal allotransplantation, and tissue engineering [4,6]. Among the various techniques currently available for airway replacement, the most appropriate one is the use of a biocompatible and rigid yet flexible tube facilitating re-epithelialization, which offers the advantages of resisting bacterial colonization and stenosis and integration with the surrounding tissues. However, adopting foreign materials for airway replacement led to issues such as airway obstruction, chronic infection, prosthesis movement, granulation tissue growth, and erosion in the major blood vessels. Moreover, implanting lyophilized, frozen, or chemically-treated non-viable tissues induced dismal functional outcomes. Tracheal allotransplantation, on the other hand, produced unsatisfactory outcomes in terms of complications such as stenosis or graft necrosis. Even immunosuppressive therapy used in anticancer treatment was ineffective. Therefore, these methods were gradually discarded. Autogenous tissue reconstruction was attempted for tissues such as Fascia Lata, skin, costal cartilage, pericardium, bowel, esophagus, and bladder. However, due to the involved complicated procedures, unfavorable outcomes were obtained, except for the reconstruction of cartilage and muscle flaps. Since the preliminary research conducted by Vacanti et al., an increasing number of studies are exploring the application of tissue-engineering technologies to achieve the formation of the epithelial cells-covered cartilaginous tube [[7], [8], [9]]. Currently, tissue engineering-based tracheal replacement is not used in cancer treatment as this procedure requires using the cells from the patients and involves a long period of several months for graft construction, as described in the book published by Grillo. Previous studies on airway transplantation did not report consistent results, due to which no consensus has so far been reached on the standard procedure for tracheal replacement [6,10,11]. Unfortunately, this promising way of research was discredited by some investigators who were accused of scientific misconduct, this leading to ethical debates [12].

Since Grillo's textbook, great achievements have been reported in the field of in-vitro tissue engineering with the use of stem cells [13]. In the mid-20th century, modern stem cell studies were initiated, which allowed for defining the basic stem cell characteristics, such as multipotentiality, self-renewal, and clonogenicity. Consequently, stem cells could be identified and classified into totipotent, pluripotent, multipotent, and progenitor cells. Later, stem cells were also cultured and differentiated into different cell types to explore their application potential in tissue engineering and the associated treatment approaches. Tissue-specific or embryonic adult stem cells are recommended for treating different human diseases. In addition, *ex vivo* tissue engineering is proposed as a tissue reconstruction approach. This approach involves culturing human cells in artificial bioreactors and then seeding them in scaffolds, which are finally implanted into the target tissue to be engineered [14].

In 2008, Birchall's group in London pioneered tissue engineering-based airway transplantation in a case of post-tuberculosis end-stage bronchomalacia [15]. In their study, mesenchymal stem cell (MSC)-derived chondrocytes and recipient epithelial cells were cultured and seeded on the decellularized trachea from a human donor inside an artificial bioreactor. Encouraging outcomes were obtained four months after the implantation. However, the approach had certain limitations, such as a lack of human trachea donors and the unsuitability of the approach for cancers due to the requirement of epithelial cells from the patients and several months for graft acquisition [16].

Therefore, later in 2011, Birchall's primary team used a bioartificial nanocomposite in place of decellularized human donor trachea. The nanocomposite was seeded on the artificial bioreactor along with autologous bone marrow mononuclear cells (MNCs) [17]. The procedure was first performed for a case of extensive relapsed cancer. After the procedure, cell-mediated wound repair, stem cell homing, graft neovascularization, and extracellular matrix (ECM) remodeling were observed. Upon the addition of a growth factor, the apoptosis and migration of the stem cells were inhibited [18]. Favorable outcomes were attained in 5 months, as observed during follow-up. In 2012, Elliott et al. pioneered tissue engineering-based tracheal replacement using stem cells in a child, achieving favorable outcomes in 2 years during follow-up [19].

Delaere et al. in Leuven conducted the first novel surgery on a patient who had developed extensive post-traumatic stenosis in 2010 [20]. The patient received tracheal allograft wrapping in the forearm fascia while undergoing immunosuppressive treatment. Progressive revascularization of the tracheal allograft and full lining with the buccal mucosa from the recipient and the respiratory epithelium from the donor could be observed. The tracheal allograft with complete blood supply was later transferred to the anatomical location when the immunosuppressive treatment was withdrawn after 4 months. Favorable outcomes were attained in 1 year during follow-up. However, the patient developed total atelectasis of the left lung, which was complicated by acute respiratory failure, in February 2014. The patient was then admitted to the Department of Thoracic Surgery once again and received transternal left pneumonectomy in 2016. The patient underwent smooth postoperative recovery, with full recovery at 30 months post-pneumonectomy and no other medical events [21]. Besides requiring immunosuppressive treatment, the above procedure is also associated with problems such as *ex vivo*-engineered airways [22].

Besides, since Grillo's textbook, great achievements have been attained in the field of *in-vitro* tissue engineering using stem cells [13]. The initiation of modern stem cell studies in the mid-20th century allowed for defining the basic stem cell characteristics, such as multipotentiality, self-renewal, and clonogenicity. Consequently, *ex vivo* tissue engineering has emerged as a candidate approach for tissue reconstruction. In this procedure, human cells are cultured and seeded into the scaffolds inside artificial bioreactors, followed by implanting the cells in the target tissue to be engineered [14].

The first tissue engineering-based airway transplantation was conducted by Birchall's group in London in 2008, for a patient who had developed post-tuberculosis end-stage bronchomalacia [15]. The MSC-derived chondrocytes and recipient epithelial cells were cultured and seeded on the decellularized trachea from a human donor inside an artificial bioreactor, and encouraging outcomes were attained four months post-implantation. The approach was, however, limited due to the lack of human trachea donors. In addition, the approach was unsuitable for cancer treatment as epithelial cells from the patients were required and graft acquisition happened in several months [16].

Therefore, in 2011, rather than using the decellularized trachea from a human donor, Birchall's primary team used a bioartificial nanocomposite, which was seeded on the artificial bioreactor along with autologous bone marrow MNCs [17]. The procedure was first applied to a case of extensive relapsed cancer. Cell-mediated wound repair, stem cell homing, graft neovascularization, and ECM remodeling were observed in the case after the procedure application. After the addition of a growth factor, the apoptosis and migration of the stem cells were inhibited [18]. Favorable outcomes were attained in 5 months during follow-up. Elliott et al. performed tissue engineering-based tracheal replacement using stem cells first time in a child in 2012 and achieved favorable outcomes in 2 years during follow-up [19].

In 2010, Delaere et al. conducted a novel surgery in Leuven for a patient who had developed extensive post-traumatic stenosis [20]. The patient received tracheal allograft wrapping in the forearm fascia while undergoing immunosuppressive treatment and exhibited progressive revascularization of the tracheal allograft and full lining with the recipient's buccal mucosa and donor's respiratory epithelium. Later, when immunosuppressive treatment was withdrawn after 4 months, the tracheal allograft with complete blood supply was transferred to the anatomical location. Within 1 year of follow-up, favorable outcomes were attained. However, the patient developed total atelectasis of the left lung complicated by acute respiratory failure in February 2014 and had to be admitted again to the Department of Thoracic Surgery. In 2016, this patient received transternal left pneumonectomy, following which smooth and full recovery occurred at 30 months post-pneumonectomy, without any other medical events [21]. Besides the necessity of immunosuppressive treatment, the procedure is associated with problems such as *ex vivo*-engineered airways [22].

## 3 Airway transplantation using an aortic matrix

The procedures used in *ex-vivo* tissue engineering are complicated and render it difficult to achieve the reconstruction of functional organs or tissues even after several attempts. Therefore, researchers propose using *in-vivo* tissue engineering, which adopts the human body as the natural bioreactor for cell growth and culture [23]. In 1997, Martinod and colleagues initiated a research program on airway transplantation using aortic grafts at the Laboratory of the Alain Carpentier Foundation, Paris [24]. In the preliminary research of these authors on tracheal replacement, the authors realized that the aorta had been neglected in this regard. The aorta offered several advantages, including a diameter close to that of the trachea, a solid structure, elasticity, and resistance to infection. However, it also had certain drawbacks, such as the risk of collapse, which was, however, preventable using a stent. In subsequent experiments, tracheal replacements with aortic autografts were performed using fresh as well as cryopreserved allografts [25,26]. In initial experiments, a transversal cervical incision was performed in 10 sheep, with resection of one anterior half-circumference of two tracheal rings and replacement via the autologous right carotid artery patch. All sheep survived postoperatively, except for the one sheep that died due to a major cervical abscess arising in the graft 16 days postoperatively [25]. Most sheep did not develop complications in the 3-year follow-up period [26].

**Fig. 1:**
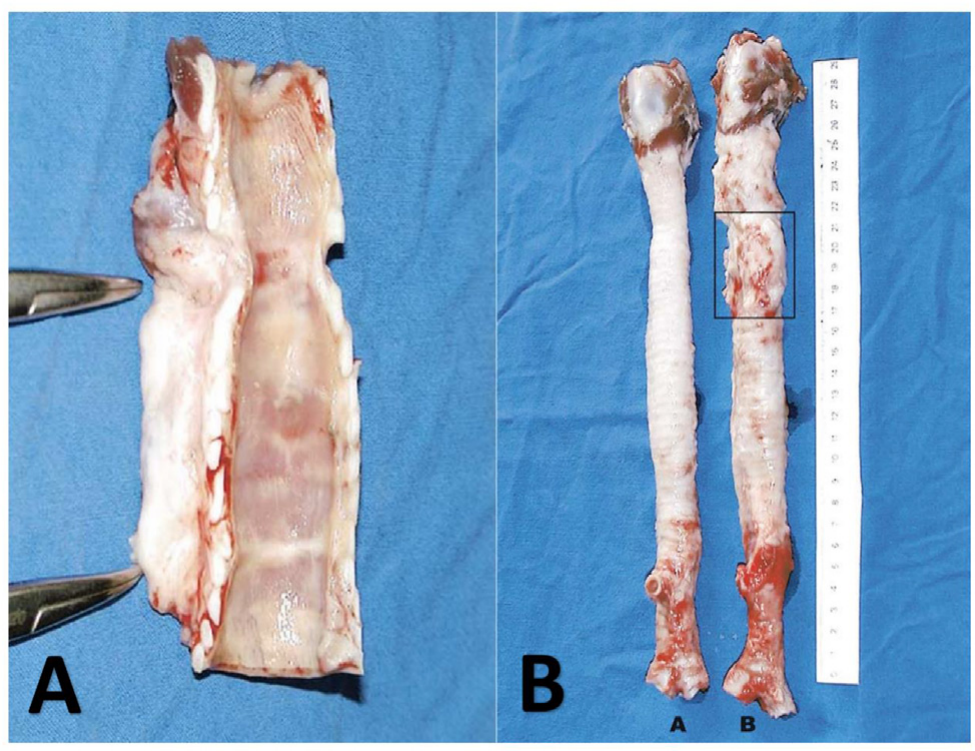
Cartilage regeneration of the grafts implanted in sheep.

The macroscopic assessment results were quite surprising. First, stenosis was not detected in those receiving stent insertion in the graft. Second, tracheal regeneration was achieved in one case, which included both cartilage and the epithelium (Fig. 1), similar to what was observed after carina replacement [27]. Prior to the experiments, Martinod and colleagues decided to use of a nitinol stent. However, this procedure was not feasible due to excessive adhesion following such stent use. Consequently, Martinod and colleagues placed a silicone stent in the graft, which could be easily removed, and no clinical consequences were observed, indicating the functionality of the regenerated cartilage. The histological analysis revealed that the epithelium had progressively regenerated from the inflammatory tissue to the mucociliary, mixed, and squamous epithelium.

One month later, the usual aortic histological structure had almost disappeared and was replaced with dense fibrosis, excessive inflammatory tissue, and scattered spots of the metaplastic, non-keratinizing, and poly-stratified squamous epithelium. The fibrosis contained immature cartilage and disorganized islands of elastic fibers. Three months later, the intensity of inflammation decreased after the appearance of the cartilage (continuous mucociliary epithelium appeared in one case). Six to twelve months later, 5–7 cartilage rings had formed due to the above-described regeneration process, along with continuous mucociliary epithelium or blended epithelium comprising squamous, secretory, and ciliated cells. Moreover, in the sample collected after 24 months, the histology had completely transformed to that of the tracheal tissue, containing regular rings of the new cartilage, the functional continuous mucociliary epithelium recovered following stent removal, and one posterior fibrous membrane [26].

A. Macroscopic image of the 24-month sample exhibiting proximal and distal anastomoses (indicated with forceps), novel cartilage rings, and posterior membrane (sample bottom); B. Macroscopic image for a comparison of the healthy trachea (A) with the grafted trachea (B) resected and three years after grafting (the rectangle indicates the grafted region). The total number of cartilage rings was similar for the two samples (50 vs. 48, respectively) [Martinod E, Seguin A, Pfeuty K et al. Long-term evaluation of the replacement of the trachea with an autologous aortic graft. Ann Thorac Surg. 2003 May; 75(5):1572-8; discussion 1578.].

The observations were similar to those in the regeneration achieved using other following epithelial destruction. The process began from the native tracheal mucosal and basal cells. At each interval, residual elastic fibers were observed in the aortic tissue. Collagen-secreting fibroblast reconstruction was observed during inflammation. In the allograft study by Martinod et al. [28], the authors implanted male sheep aorta into female sheep trachea in six to reveal whether the cartilage was derived from recipient cells or aortic cells animals and identified SRY genes within the new cartilage. Using the marker for type 2 collagen, the authors discovered that the new structures formed in the aortic graft were cartilage structures.

The aortic graft necrosis detected in each animal was attributable to the use of a prosthesis to isolate the homograft from the blood supply and the adjacent tissues. Importantly, the bronchoscopic examinations revealed no graft rupture, anastomotic leakage, and stenosis, although one animal developed an infection in the tracheal granuloma after 1 month. The histology results were further intriguing [25]. Samples were collected at various time points postoperatively to reveal the histological changes in the engrafted aortic tissue. A strong inflammatory reaction occurred after 1 month after replacing the aortic tissue, and no epithelial organization was detected. Meanwhile, disorganized elastic fibers were detected inside the aortic graft, and the number of these fibers exhibited a decreasing trend during the follow-up period. Several remnants existed even after 24 months. Three months later, the inflammatory reaction continued to be dominant, with the presence of metaplastic, non-keratinizing squamous epithelium adjacent to anastomoses. Furthermore, by the time interval of three to six months, a continuous epithelium had formed at the graft center (poly-stratified) and adjacent to the anastomoses (mucociliary), indicating the completion of the regeneration process. In the 1–6 months interval, no cartilage differentiation was evident in the graft. Six months later, immature cartilage islands were observed adjacent to anastomoses, while the cartilage at the graft center had not regenerated yet. In the sheep that underwent stent removal after 9 months followed by sacrificing after 24 months, both continuous cartilaginous and mucociliary epithelium had formed [27].

**Fig. 2:**
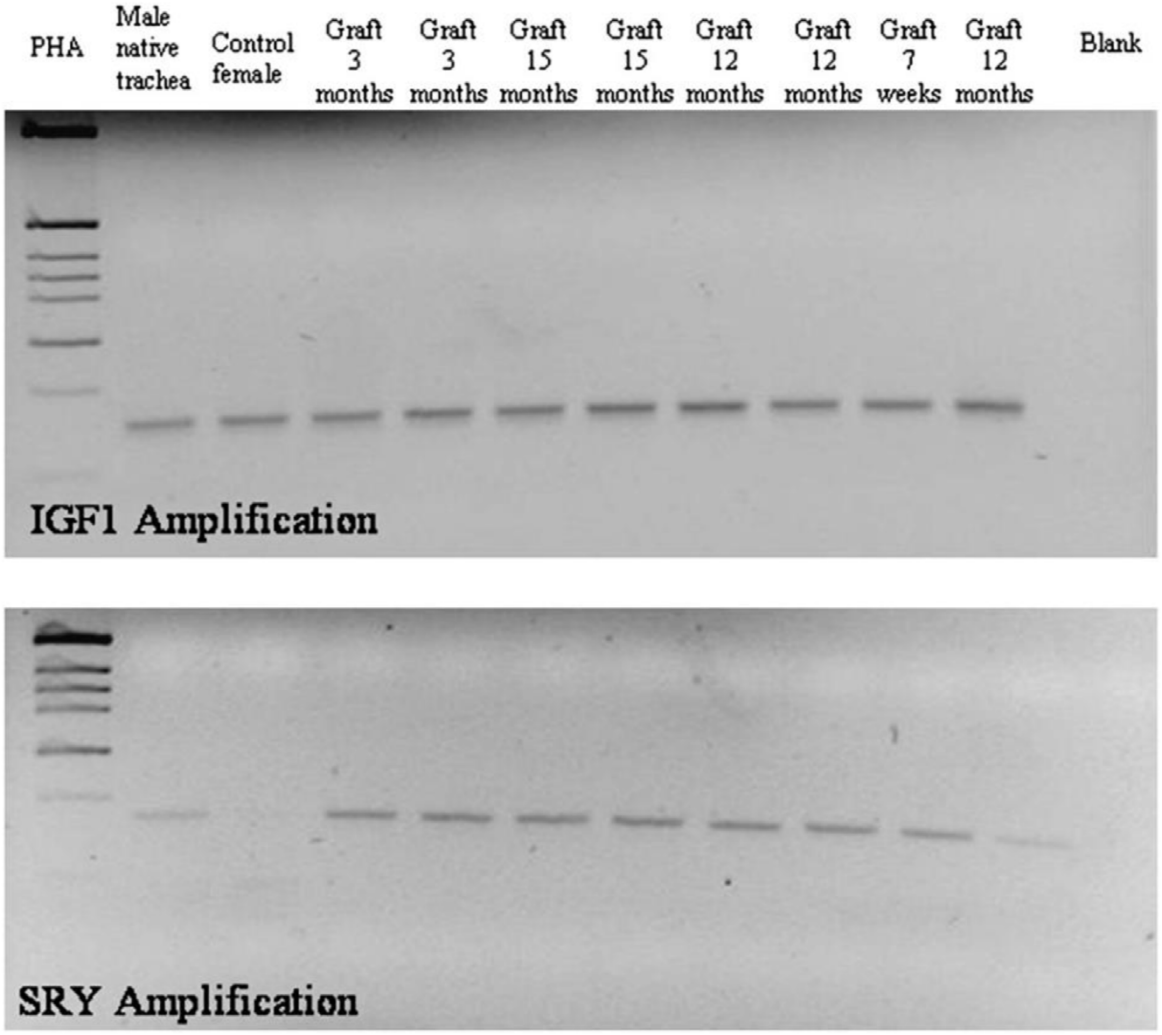
SRY genes in the new cartilage.

According to the PCR results, SRY was amplified in the male samples and not in the female ones or the new cartilage. This suggested that the new cartilage was derived from recipient cells rather than aortic cells [28] (Fig. 2). The follow-up studies led by Seguin et al. and Radu et al. (Martinod's group) [29,30] also revealed that the process was not derived from aortic cells. Moreover, chondrocytes could not migrate, and the new cartilage was far away from the native trachea, rendering native cartilage regeneration impossible. In addition, it was hypothesized that the regeneration was derived from local cells, mesenchymal cells, or probably the bone marrow-derived circulating stem cells since it was illustrated for additional organ repair, as demonstrated by Seguin et al. [31] in rabbit models.

Amplification bands of the SRY and IGF1 genes on 2% agarose gel. PHA 5 phytohemagglutinin [Makris D, Holder-Espinasse M, Wurtz A et al. Tracheal replacement with cryopreserved allogenic aorta. Chest. 2010 Jan; 137(1):60-7.].

Cryopreserved aortic allograft is probably the superior choice for human applications owing to its wide availability in tissue banks, no requirement for immunosuppression, and the possibility of permanent storage. According to Martinod et al., functional tissue regeneration could be achieved using cryopreserved aortic allografts in tracheal replacement rather than using glutaraldehyde-mediated or decellularized aortic grafts. Notably, the regeneration occurred in a pattern identical to that observed for fresh allografts. These findings were verified by Martinod et al. [32]. As hypothesized by the Seguin et al. (Martinod's group) [31], the airway healing achieved after biological scaffold replacement was attributable to airway regeneration and approximation.

## 4 Experimental studies to initial human applications

In 2004, Azorin et al. replaced a tumoral trachea (length, 7 cm) with a silicone stent-supported aortic infra-renal autograft [33]. This was the only human application using an aortic autograft, cryopreserved aortic allografts having demonstrated their superiority by preventing harvesting from the patient himself [34].

**Fig. 3:**
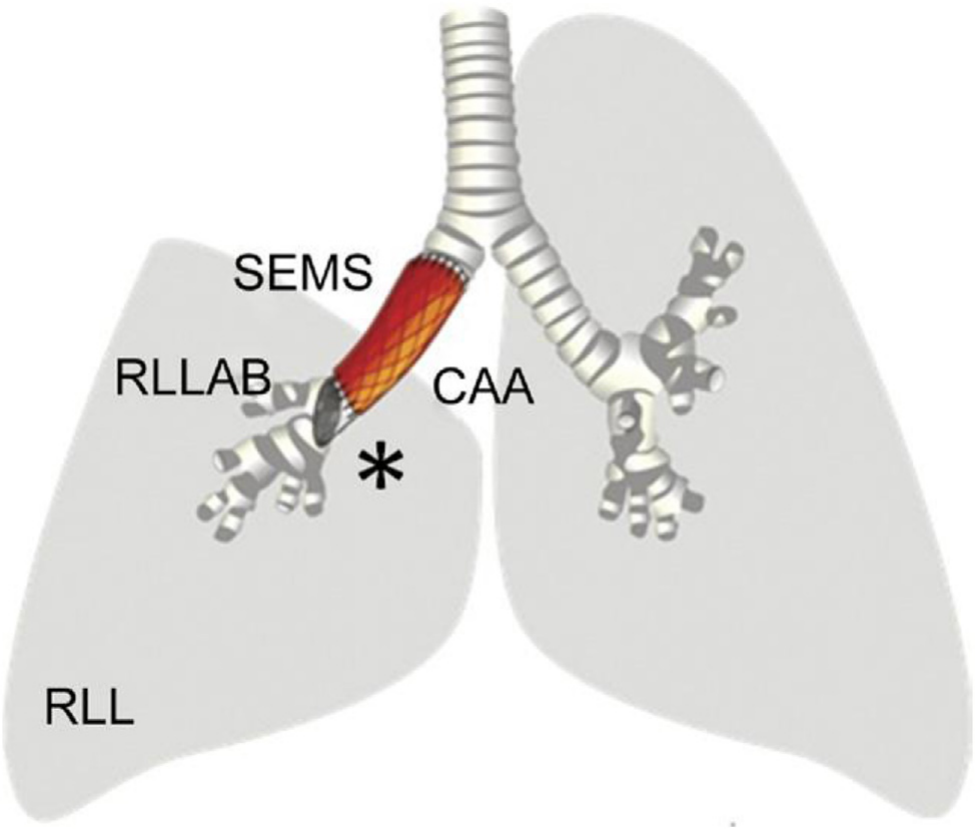
Sketch map depicting the procedure.

*Note: The preserved right lower lobe (RLL) and the cryopreserved aortic allograft (CAA, depicted in red)* supported *by the customized, fully-covered, conical, self-expanding, metallic stent (SEMS, depicted in black) comprised the oblique distal segment (∗) for allowing RLL bronchial ventilation, including the right lower lobe apical bronchus* (*RLLAB) [*Martinod E, Radu DM, Chouahnia K et al. Human transplantation of a biologic airway substitute in conservative lung cancer surgery. Ann Thorac Surg. 2011 Mar; 91(3):837-42*.]*.

In 2009, Martinod et al. applied the procedure to a 78-year-old patient, who was a high-risk case and had developed extensive right bronchopulmonary cancer, for which the patient had received pretreatment with chemotherapy [35] (Fig. 3). The patient was subjected to upper bilobectomy plus lymph node dissection, which completely resected the lung cancer. However, primary end-to-end bronchial anastomosis was not feasible in the mobilization procedures. Therefore, bronchial continuity was restored by interposing a certified tissue bank-derived stent-supported cryopreserved aortic allograft, and the lower lobe was preserved. The patient was followed up for 1 year after surgery, and in this period, favorable outcomes in terms of the functionality of the reimplanted lower lobe and no stent-related or cryopreserved aortic allograft-associated complications. In this case, recovery to daily life activities was smooth, with a high quality of life in terms of health [34]. Two additional human cases were successfully performed by the same group in order to avoid a definitive tracheostomy for complex laryngotracheal stenoses refractory to conventional therapy [32].

## 5 ‘TRACHEO BRONC-ART’ and ‘TRITON-01’ studies

Achievements related to the field of airway transplantation are associated with critical ethical and scientific controversies [35,36]. Therefore, the International Society for Cell Therapy, along with certain ethics experts and regulators, proposed specific recommendations for human airway bioengineering in 2014 [37]. The associated research was initiated in 1997, and since then, the Martinod and colleagues have closely followed the international scientific and ethical principles of surgical innovation [38].

In order to establish whether airway bioengineering based on implanting stented aortic matrices was clinically feasible, Martinod et al. [39] conducted an uncontrolled single-center-based cohort study, in which cases with proximal lung cancer that required pneumonectomy or those having end-stage tracheal lesions (NCT01331863) were included. The study was conducted in Paris, France, between October 2009 and February 2017. Each included case was followed up until November 2, 2017, with 90-day mortality used as the primary endpoint and 90-day morbidity used as the secondary endpoint of the study. In total, 20 cases aged 24–79 (average age, 54.9) years were included in the study, including 13 males [65%]. Among these 20 cases, 13 cases received bronchial (n = 7), tracheal (n = 5), and carinal (n = 1) transplantation. The remaining 7 cases did not receive airway transplantation due to exploratory thoracotomy alone, unavoidable pneumonectomy, and medical contraindication, while bilobectomy and lobectomy were feasible. Among the 20 included cases, the overall 90-day mortality rate was 5% (only one patient who received carinal transplantation died). Among those who received bronchial and tracheal reconstruction, no death was reported during the 90 days. Among the cases who underwent airway transplantation, 30.8% of the cases reported major 90-day morbidity events, including acute lung edema, laryngeal edema, atrial fibrillation, and acute respiratory distress syndrome. In these patients, surgery-related adverse events were not reported. Stent was removed at an average of 18.2 months postoperatively. In the median follow-up duration of 3 years and 11 months, 10 among the 13 cases (76.9%) remained alive. Among these survivors, 8 (80%) cases reported normal breathing via the new airways following stent removal. Epithelial regeneration and *de novo* cartilage generation were observed inside the aortic matrices of the recipient's cells.

The above results indicated that stented aortic matrices-based airway bioengineering is feasible for complicated bronchial and tracheal reconstruction. It is recommended that rather than establishing rules, prospective surgical protocols should be formulated prior to introducing this approach to the clinic, regardless of the international recommendations.

**Fig. 4:**
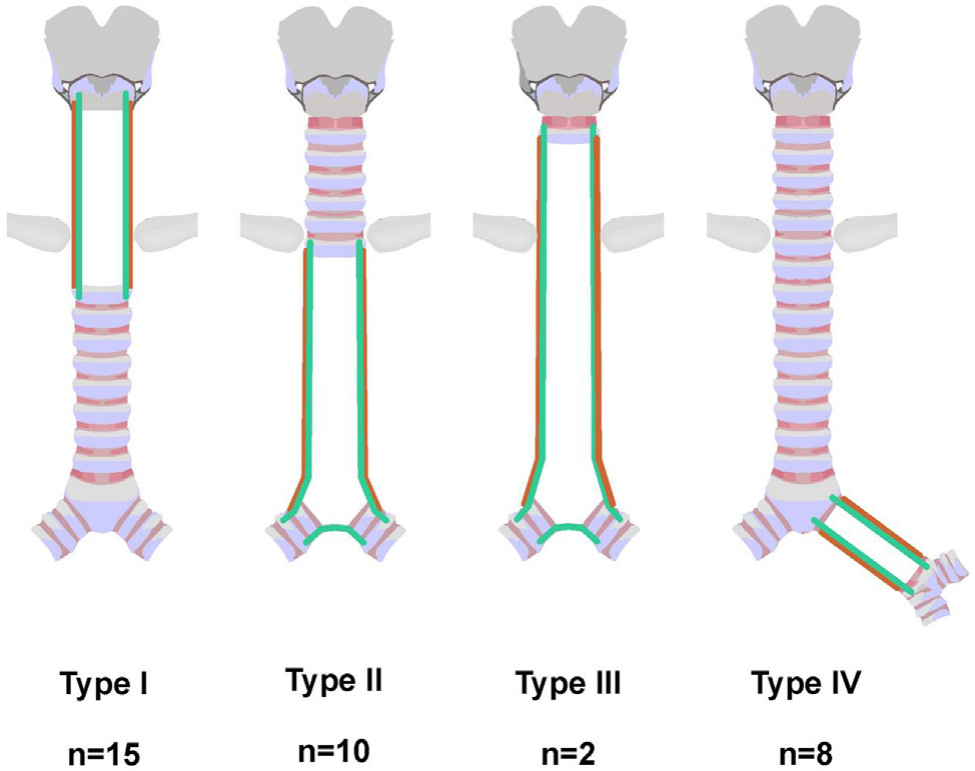
Lesions and surgical treatment types.

Martinod et al. [40] then reported the long-duration follow-up outcomes of the TRITON-01 study (NCT04263129). In total, 35 cases received airway replacement due to malignant and benign lesions (n = 29 and 6, respectively) between October 2009 and October 2021. In these cases, a mortality rate of 2.9% and a morbidity rate of 22.9% were reported 30 days after surgery. The patients were followed up for 1–133 (median, 29.5) months, and only 27 survived. Death due to implanted bioprosthesis did not occur in these patients. Meanwhile, 18 cases (52.9%) developed stent-induced granulomas that required bronchoscopic treatment. In addition, 8 patients among the 35 cases (28.6%) survived without a stent. The Kaplan-Meier analysis revealed the 2-year and 5-year survival rates of 88% and 75%, respectively. These results of the TRITON-01 study indicate the feasibility of stented aortic matrices-based airway replacement for routine care (Fig. 4).

Classification of lesions according to their topology and the corresponding surgical treatment. Schematic diagram: an upper (cervical) tracheal lesion extending to the middle trachea or cricoid cartilage, a middle-lower (thoracic) tracheal lesion extending or not extending to the upper bronchus and/or the main bronchi and/or carina, then to (sub) the entire trachea, and eventually to the bronchus, was observed in 15, 10, 2, and 8 patients, respectively. Types I-IV included the following respectively: 10 cases of thyroid/parathyroid cancer and 5 cases of end-stage post-intubation tracheal stenosis cases; 10 cases of extended tracheobronchial cancer; 2 cases of total/subtotal tracheal and carina invasion (due to large adenoid cystic cancers); 7 cases of lung cancer; 1 case of end-stage bronchial stenosis [Martinod E, Radu DM, Onorati I et al. Airway replacement using stented aortic matrices: Long-term follow-up and results of the TRITON-01 study in 35 adult patients. Am J Transplant. 2022 Dec; 22(12):2961–2970.].

As verified in the present review, stented aortic matrices-based airway replacement is applicable in routine care. However, further studies are necessary to determine whether this innovative approach is generalizable. Advancements in translational research for investigating the regeneration phenomenon has enabled the optimization of cartilage formation inside the cryopreserved aortic allografts and the evaluation of novel bioengineered and/or 3D-printed aortic graft types that could be used commercially and extensively. The prospective open-label randomized TRITON-02 (Tracheal Replacement In ThyrOid caNcer) study (Protocole Hospitalier de Recherche Clinique K2019) is planned to be conducted soon at 20 French centers to compare this innovative approach to other standard treatments such as chemotherapy and radiotherapy [41].

## 6 Mesenchymal stem cells in tracheal regeneration

In 2009, to further elucidate tracheal regeneration, Seguin et al. (Martinod's group) [28] proposed the involvement of stem cells. According to the conventional opinion on the differentiation of adult stem cells, stem cell progeny develops linearly and irreversibly. This notion limits stem cell propensity and fate to just one germline [42]. According to the novel stem cell differentiation evolution theory, a stem cell progeny exhibits graded differentiation, resulting in a gradual restriction of the daughter cells possessing transgerm potential. One precedented belief is that the marrow adipocyte clonal strains are directed for bone formation, while chondrocytes are dedifferentiated into the osteogenic lineage [43]. This theory explains, as also indicated by recent research, the neurogenic potential of MSCs, the transformation of neurogenic precursors to blood and muscle cells, and the induction of the transformation of hematopoietic stem cells into hepatocytes and, therefore, represents the beginning of a paradigm shift [44]. The above-stated findings suggest that microenvironmental signals significantly affect cell fate. Consistent with this, the fate of pluripotent stem cells, which was initially considered to be limited to the lineage hierarchy, may be altered and these cells may be *trans*-differentiated [4].

**Fig. 5:**
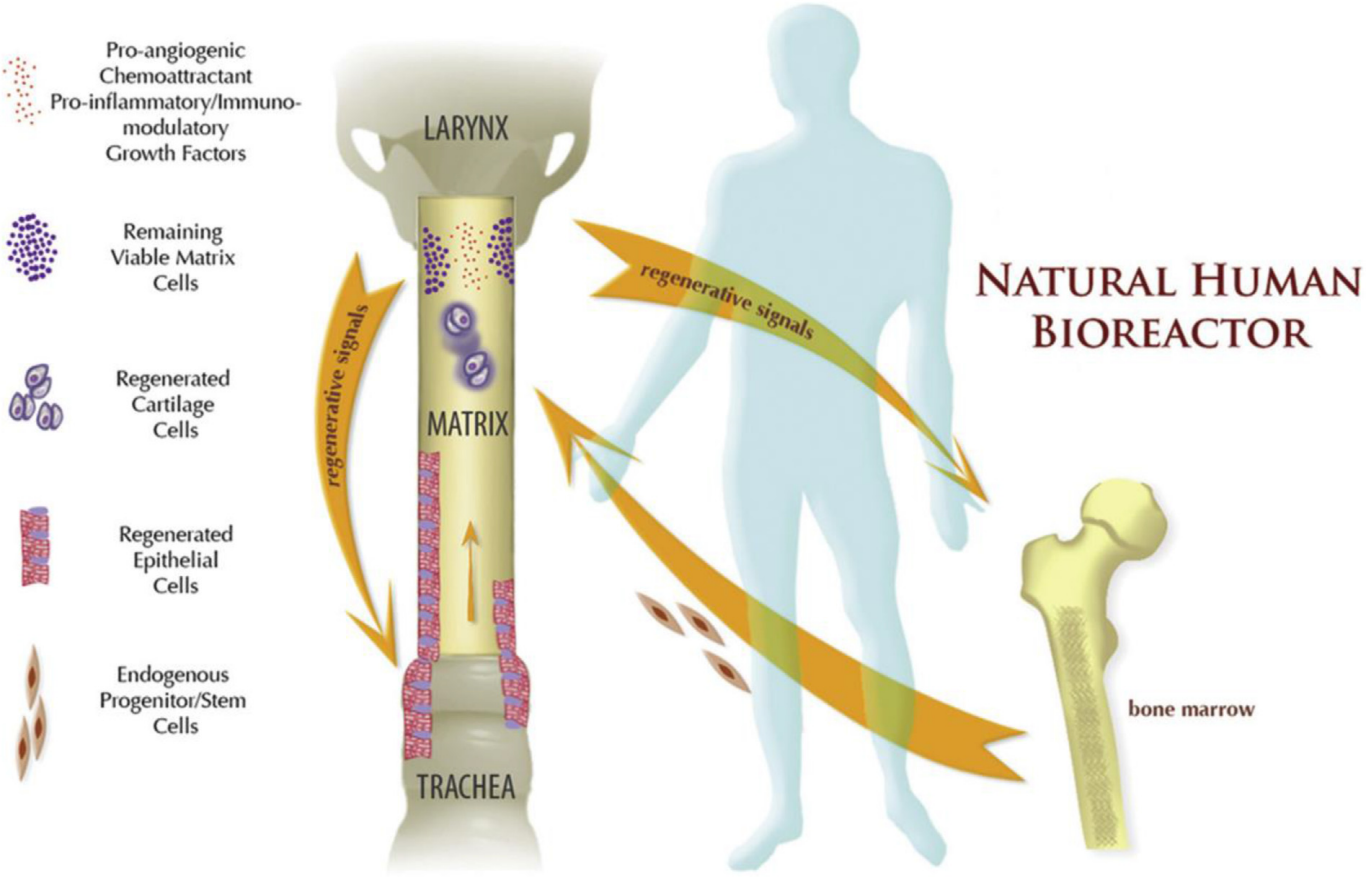
The mechanisms underlying *in vivo* airway regeneration based on tissue engineering.

According to the assumption of Seguin et al. (Martinod's group) [31], the environment significantly affects the induction of tissue transformation, resulting in the formation of structures as complicated as those of the epithelium and cartilage. The authors conducted the PCR assay and chromosome Y identification for the female sheep that received the aortic tissue from male sheep and accordingly suggested that cartilage cells were derived from the recipient. Therefore, Makris assumed that the recipient's multipotent MSCs induced upon environmental signals exhibited colonization on the graft and differentiation to chondrocytes (Fig. 5).

When the injured airway was replaced, the remaining living aortic matrix cells produced growth factors, chemoattractant, proangiogenic, and proinflammatory/immunomodulatory factors, which promoted the general and local stem/progenitor cell homing. Using the human body as a natural bioreactor, epithelial and cartilage regeneration was achieved inside the matrix of the recipient cells [Martinod E, Paquet J, Dutau H et al. In Vivo Tissue Engineering of Human Airways. Ann Thorac Surg. 2017 May; 103(5):1631–1640.].

**Fig. 6:**
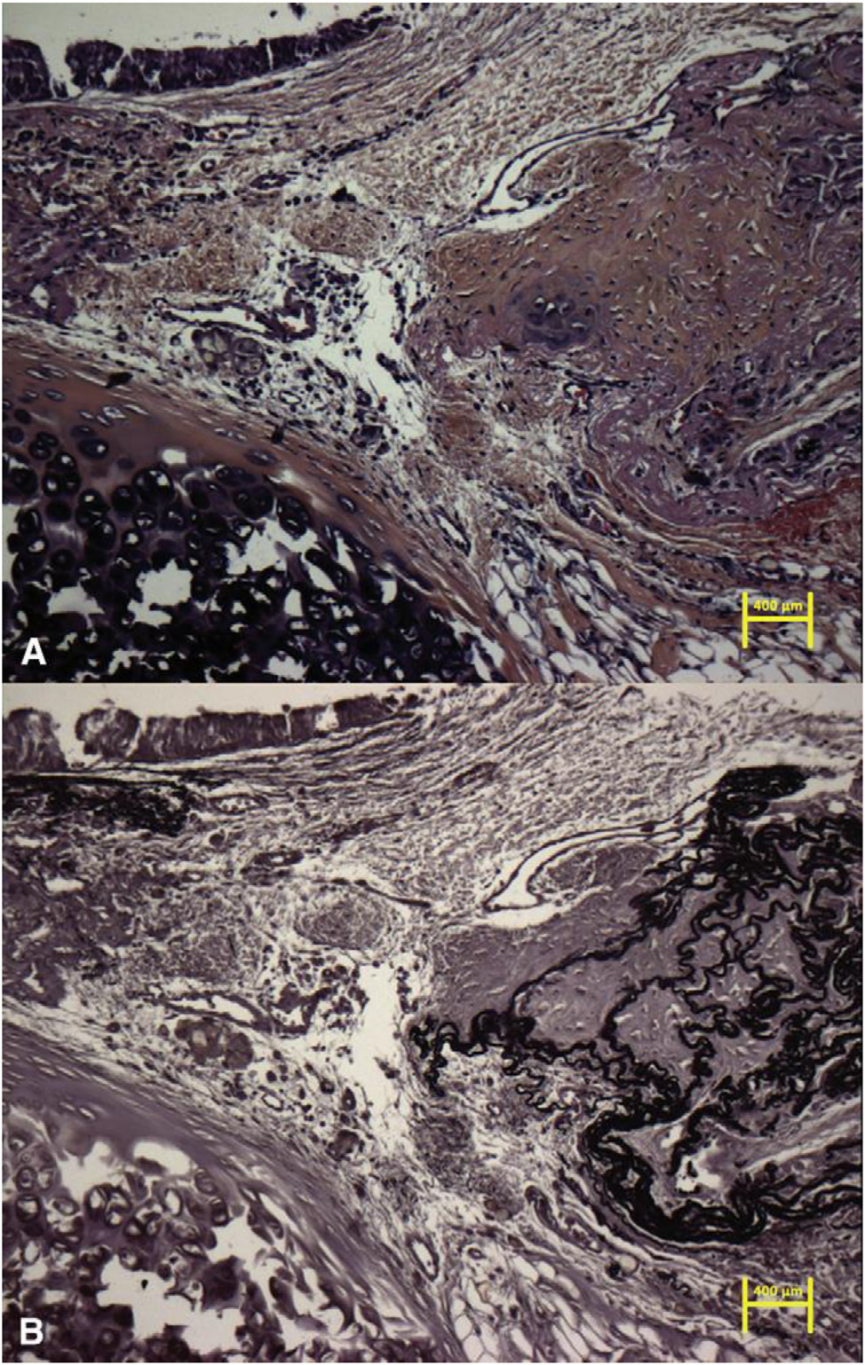
Graft in the injection group 3 months later.

In order to further explore the effects of MSCs on airway regeneration, Seguin et al. (Martinod's group) [31] subjected three groups of rabbits (groups 1–3) to tracheal replacement, autologous green fluorescent protein (GFP)-labeled MSC transplantation plus tracheal replacement after 3 months, and tracheal replacement plus autologous GFP-labeled MSC injections at 0, 10, and 21 days postoperatively, respectively. Fluorescence *in situ* hybridization (FISH) for EFGFP was conducted to assess the contributions of GFP-labeled MSCs to the structures inside the allograft after the cells were injected intravenously post-aortic allografting or the labeled MSCs were transplanted. Beginning from Day 21 to the end of the study, MSC homing and migration into the graft were observed. The EFGFP epithelial cells were detected in the rabbits that received the GFP-labeled MSCs injected intravenously or transplanted from the bone marrow. In the rabbits that received the transplant, most epithelial cells were labeled with GFP, while moderate numbers of EFGFP-labeled cells were detected in rabbits injected with labeled cells. The mucociliary differentiation regions, which were GPF-positive, appeared over time, mainly at the remodeled tissue edges that grew to the graft center. Positively-labeled cells were observed across connective tissues at the depth of the graft. The cartilage was moderately positive, mainly at the activating border, which was also verified in the injection groups 6 and 9 months later (Fig. 6).

A. Hematoxylin–eosin–saffron stain (initial magnification 310) depicting the location of anastomosis, in which new cartilage was formed and the epithelium was regenerated. B. Orcein stain (initial magnification 310) displaying the elastic fibers inside the cartilage island [Seguin A, Baccari S, Holder-Espinasse M et al. Tracheal regeneration: evidence of bone marrow mesenchymal stem cell involvement. J Thorac Cardiovasc Surg. 2013 May; 145(5):1297–1304. e2.].

The tracheal regeneration is also explained by proposing the existence of stem cell ‘‘niches’’, which may induce the total mucociliary epithelium following tracheal damage [45]. However, no study to date has reported the specific stem cells separated from the trachea that induce cartilage regeneration. Seguin et al. (Martinod's group) [31] assumed that the activation of multipotent MSCs induced in the recipient upon environmental signals led to graft colonization and tracheal regeneration.

Considering the great GFP-labeled MSC pools, Seguin et al. (Martinod's group) [31] suggested a higher probability of such cells migrating into the graft. The specialized respiratory epithelium covering the anastomotic line, which originates from the surrounding healthy cell overgrowth and the resulting functional intraluminal lining, is the best consequence following any airway surgery. Certain scholars have stated that secretory and basal cell growth, differentiation, and migration to ciliated cells induced epithelial regeneration following experimental tracheal epithelial injury similar to the epithelial generation observed in the fetal stage [46]. A similar hypothesis was proposed, which suggests that the mucus cells and basal cells migrate to the injured region and form the mucociliary epithelium following the transitory stage of the metaplastic epithelium [47]. Later, Seguin et al. (Martinod's group) focused on tracking the labeled MSCs *in vivo* to study cartilage regeneration in the aortic allograft. However, mature cartilage was not detected in the graft and rather in the islands at the grade (or anastomosis) edge [31].

Recent studies have reported the presence of testosterone and estrogen receptors on stem cells, which suggests the potential roles of these two in modulating cell functions [48]. In addition, sex hormones might play critical modulatory roles in immune/inflammatory responses, altering cell engraftment, recruitment, and mobilization. Experiments on rabbits and pigs have produced unsatisfactory outcomes. Similar experiments were conducted with male animals, with sheep protocols implemented in juvenile female sheep. It is also possible that graft function induces maturation. Grafts were reportedly not that potent in small animal models (rabbit), as confirmed by the coughing, thick secretion, and incapability of aspiration observed in these animals. Such reduced functionality may be related to the reduced maturation in certain specific biomimetic environments that are capable of inducing cell functions and phenotypes [49].

## 7 Future direction

Future research in this regard could focus on accelerating *de novo* cartilage generation for early stent removal, identifying the mechanism underlying airway regeneration using cryopreserved aortic matrices, assessing the long-term quality-of-life in the cases undergoing transplantation, and evaluating the clinical application based on multicenter studies, particularly for the cases developing lung cancers that require pneumonectomy or those having end-stage tracheal lesions.

## 8 Conclusions

Functional tissue regeneration is achieved after tracheal replacement using cryopreserved aortic allografts, and this regeneration follows a pattern identical to that observed for fresh allografts. Nonetheless, whether we are reaching any closer to the Holy Grail of airway transplantation by using cryopreserved aortic allografts remains unknown so far [50]. However, it is confirmed that cryopreserved aortic allografts offer several advantages, such as easy availability in tissue banks, long-term storage, and no requirement for immunosuppression. Moreover, the use of an optimal matrix accelerates *in situ* tissue engineering in animal models, which applies to both malignant and benign lesions. Therefore, this approach has the potential for clinical application in the treatment of patients with extensive tracheal cancer.

## Funding

Natural Science Foundation of Jilin Province (No.YDZJ202301ZYTS456); Education Department of Jilin Province (No.JJKH20231207KJ); Youth Development Fund of the First Hospital of Jilin University (No.04046910001).

## Declaration of competing interest

The authors declare the following financial interests/personal relationships which may be considered as potential competing interests:

Shixiong Wei reports financial support was provided by Natural Science Foundation of Jilin Province. Shixiong Wei reports financial support was provided by Education Department of Jilin Province, China. Shixiong Wei reports financial support was provided by Youth Development Fund of the First Hospital of Jilin University.
